# 16S rRNA Gene Amplicon Profiling of Baby and Adult Captive Elephants in Thailand

**DOI:** 10.1128/MRA.00248-20

**Published:** 2020-06-11

**Authors:** Sangam Kandel, Supaphen Sripiboon, Piroon Jenjaroenpun, David W. Ussery, Intawat Nookaew, Michael S. Robeson, Thidathip Wongsurawat

**Affiliations:** a Department of Biomedical Informatics, College of Medicine, University of Arkansas for Medical Sciences, Little Rock, Arkansas, USA; b Department of Bioinformatics, University of Arkansas at Little Rock, Little Rock, Arkansas, USA; c Department of Large Animal and Wildlife Clinical Science, Faculty of Veterinary Medicine, Kasetsart University, Kamphaeng Saen Campus, Nakhon Pathom, Thailand; University of Delaware

## Abstract

Here, we present a 16S rRNA gene amplicon sequence data set and profiles demonstrating the bacterial diversity of baby and adult elephants from four different geographical locations in Thailand. The dominant phyla among baby and adult elephants were *Bacteroidetes*, *Firmicutes*, *Proteobacteria*, *Kiritimatiellaeota*, *Euryarchaeota*, and *Tenericutes*.

## ANNOUNCEMENT

Of the three extant elephant species, the Asian elephant (Elephas maximus) is widely distributed throughout South and Southeast Asia. Based on direct observation, elephants are generalist herbivores which can feed on a variety of plant species and have seasonal variation in their dietary composition (1–3). Monitoring feeding behavior and selection of appropriate enrichment for captive elephants are critical components for achieving effective captive breeding programs. Understanding the microbial composition and diversity of the elephants could provide valuable information for elephant welfare assessment, help improve feeding, and prevent different diseases. This is the first observation of fecal microbial diversity among baby and adult elephants from different regions of Thailand.

Fecal samples from four baby elephants (three male and one female, aged 5 to 6 years) and four female adult elephants (aged 18 to 30 years) were collected from Lampang (18.276028N, 99.472361E), Chonburi (13.259278N, 101.152222E), Kanchanaburi (14.076584N, 99.424646E), and Ratchaburi (13.479511N, 99.644179E), Thailand (see Table 1 for details). Approximately 5 to 10 g of fecal samples were collected from domestic habitats and were placed into collection tubes containing a nucleic acid stabilizer (catalog number R1103; Zymo Research, USA). DNA extraction was performed at the Faculty of Veterinary Medicine, Kasetsart University, Thailand, using ZymoBIOMICS DNA kits (catalog number D4304; Zymo Research). Purified DNA samples were shipped from Thailand to the United States for 16S rRNA gene amplification and sequencing at Argonne National Labs.

**TABLE 1:** Details of all samples used in this study

Sample name	Data for elephants:				Collection date (yr-mo-day)	SRA accession no.	No. of raw sequencing reads	No. of quality-filtered reads
	Title	Location^*a*^	Age (yrs)	Sex				
EMD01LP_Ad	Adult from Lampang	Lampang	30	Female	2017-08-21	SRR9934021	57,976	44,463
EMD02LP_Ba	Baby from Lampang	Lampang	6	Male	2017-08-21	SRR9934022	46,391	37,444
EMD02SPT_Ad	Adult from Chon Buri	Chon Buri	18	Female	2017-06-21	SRR9934019	55,102	46,224
EMD03SPT_Ba	Baby from Chon Buri	Chon Buri	5	Male	2017-06-21	SRR9934020	51,815	41,736
EMD02EW_Ad	Adult from Kanchanaburi	Kanchanaburi	18	Female	2017-07-10	SRR9934026	56,317	47,250
EMD01EW_Ba	Baby from Kanchanaburi	Kanchanaburi	6	Male	2017-07-10	SRR9934025	75,552	62,782
EMD07RA_Ad	Adult from Ratchaburi	Ratchaburi	25	Female	2017-08-25	SRR9934024	79,074	65,951
EMD05RA_Ba	Baby from Ratchaburi	Ratchaburi	5	Female	2017-08-25	SRR9934023	44,933	37,509

a All locations are in Thailand.

The 16S rRNA gene was amplified and subsequently sequenced using a MiSeq sequencing platform (Illumina, Inc., USA) by generating paired-end reads from libraries with 250-bp inserts following the Illumina Earth Microbiome Protocol (4). The 16S rRNA V4 region was amplified using the barcoded primer set 515FB (5′-GTGYCAGCMGCCGCGGTAA-3′) and 806RB (5′GGACTACNVGGGTWTCTAAT-3′) (5).

Microbiome analysis was done using QIIME 2 version 2018.11 (6). Raw sequencing reads were first imported in QIIME 2 using the q2-import plugin and were demultiplexed using the q2-demux plugin. DADA2, via the q2-dada2 plugin, was used to generate amplicon sequence variants (ASVs)/exact sequence variants (ESVs) (7), perform quality filtering, and remove both phiX and chimeric sequences (8). Microbial taxonomy was assigned to the ASVs using a naive Bayes classifier trained on the Silva 132 99% operational taxonomic unit (OTU) reference sequences (9). Microbial taxonomy was trained on the amplicon region of interest (10) using the q2-feature-classifier classify-sklearn plugin (11).

**FIG 1:**
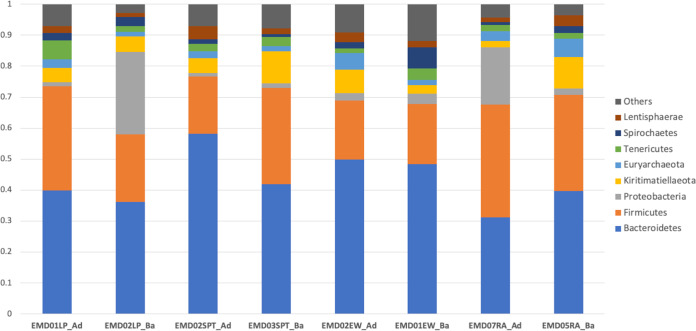
Bar chart of microbial diversity among baby and adult elephants based on 16S rRNA gene amplicon sequencing. Each bar represents the relative frequency of each microbial phylum in each sample. The top eight dominant phyla are shown.

A total of 467,160 raw reads were generated from 8 samples after demultiplexing, and 383,359 of the quality-filtered reads were used to generate 2,144 ASVs. On average, among the baby elephants, 97.4% of the reads were classified as *Bacteria*, while 2.6% were classified as *Archaea*, and among the adult elephants, 96.6% of the reads were classified as *Bacteria*, while 3.3% were classified as *Archaea*. The dominant bacteria were of the phyla *Bacteroidetes*, *Firmicutes*, *Proteobacteria*, *Kiritimatiellaeota*, *Euryarchaeota*, and *Tenericutes*, as shown in Fig. 1.

### Data availability.

The 16S rRNA gene amplicon data set was deposited in GenBank under the SRA accession numbers SRR9934019, SRR9934020, SRR9934021, SRR9934022, SRR9934023, SRR9934024, SRR9934025, and SRR9934026 (BioProject number PRJNA558043).
